# Simultaneous Blood Flow Measurement and Dermoscopy of Skin Lesions Using Dual-Mode Dermascope

**DOI:** 10.1038/s41598-018-35107-x

**Published:** 2018-11-16

**Authors:** Sean M. White, Manuel Valdebran, Kristen M. Kelly, Bernard Choi

**Affiliations:** 1 Beckman Laser Institute and Medical Clinic, University of California, Irvine, California 92612 USA; 2 Department of Dermatology, University of California, Irvine, California 92697 USA; 3 Department of Surgery, University of California, Irvine, California 92697 USA; 4 Edwards Lifesciences Center for Advanced Cardiovascular Technology, University of California, Irvine, California 92697 USA; 5 Department of Biomedical Engineering, University of California, Irvine, California 92697 USA

## Abstract

Dermascopes are commonly utilized for the qualitative visual inspection of skin lesions. While automated image processing techniques and varied illumination strategies have been used to aid in structural analysis of lesions, robust quantification of functional information is largely unknown. To address this knowledge gap, we have developed a compact, handheld dermascope that enables real-time blood flow measurements of skin during conventional visual inspection. *In-vitro* characterization demonstrated that the dermascope is capable of quantifying changes in flow across a physiologically relevant range even when used in a handheld manner with clinic lighting and dermascope LEDs on. In a small pilot clinical study, we demonstrated the dermascope’s ability to detect flow differences between two distinct lesion types.

## Introduction

Dermoscopy, the use of a handheld device with a magnifying lens and light source, is commonly used in the field of dermatology to aid in the visual inspection of suspicious skin lesions. Dermoscopy has been shown to improve diagnostic accuracy of melanocytic and nonmelanocytic pigmented lesions compared to the unaided eye, and can specifically aid the diagnosis of melanoma, basal cell carcinoma, and squamous cell carcinoma^1–3^. Timely and accurate diagnosis of such lesions is critical due largely to their particularly high incidence rate and potential morbidity. Non-melanoma skin cancer is the most common cancer in the US with an estimated 3.5 million cases each year, and although mortality rates are relatively low, it represents a significant burden to the healthcare system^4,5^. Melanoma has an estimated five-year mortality rate as high as 23%^6^ depending on lesion stage. Although dermoscopy aids inspection of lesions, the subjectivity and difficulty in interpreting magnified lesion images commonly necessitates biopsy of suspicious lesions for histopathological inspection, and this practice is still viewed as a diagnostic gold standard.

To help overcome the reliance on biopsies, diagnostic methodologies which attempt to standardize the analysis of dermascope images have been used to assess pigmented lesions. These include the ABCD rule and the seven-point check list, which rely on color and structural information to characterize lesions^7–9^. Although these tools offer a degree of quantification and objectivity to lesion assessment, their accuracy and general applicability has been questioned^10,11^. Several commercial dermascopes have been developed to aid in objective and quantitative assessment of acquired images, including MoleMax and SolarScan. These devices use an embedded camera to acquire images and software to aid in image analysis^12^.

Dermascopes with functionality beyond reliance on simple white-light illumination have also been explored to aid in diagnosis. Most commonly, cross-polarized illumination dermascopes have been used to reject superficially reflected and scattered light and thus aid in visualization of subsurface features^13^. To assist in the visualization of tissue structures within lesions that exhibit varying spectral characteristics (predominantly absorption), multispectral dermascopes have also been investigated. These devices inspect a region using different spectral bandwidths of light that can improve visualization of lesion texture, blood vessel morphology, and melanin location^2,14–17^. Commercial embodiments include the SIAscope and MelaFind, which use multispectral information to aid in identifying morphological features^18,19^.

Despite these advancements in dermoscopy, the techniques and devices described above, all rely solely on structural information to aid in diagnosis. Functional information, such as tissue perfusion, may supplement this structural information to provide a more robust characterization or diagnosis of inspected lesions. For example, it is well known that tumor progression is closely accompanied by angiogenesis as additional blood flow is needed to meet the metabolic demands of neoplastic tissue^20,21^. It has been shown histologically that the density of microvasculature is increased significantly in squamous cell and basal cell carcinomas relative to normal skin^22^. This finding has been further supported using laser Doppler perfusion imaging, which has demonstrated that blood flow in skin tumors is significantly greater than the surrounding skin and that varying degrees of skin blood flow may aid in differentiating tumor type^23,24^.

To that end, we have developed a handheld dermascope that enables the user to perform standard magnified analysis of skin lesions simultaneously with skin blood flow measurements. This was achieved by integrating a compact laser speckle contrast imaging (LSCI) system into a custom-built dermascope (called the LSCI-dermascope). While LSCI is a flexible technique for quantifying cutaneous blood flow, clinical adoption is hindered by the fact that commercial LSCI systems are relatively large and bulky, do not provide simple registration of inspected lesions with collected blood flow maps, and suffer from measurement artifact due to movement^25,26^. We have designed the LSCI-dermascope to overcome these limitations and facilitate the practical integration of perfusion assessment into a typical dermatology workflow.

## Results

### Quantification of LED/room light interference on LSCI measurements

**Figure 1:**
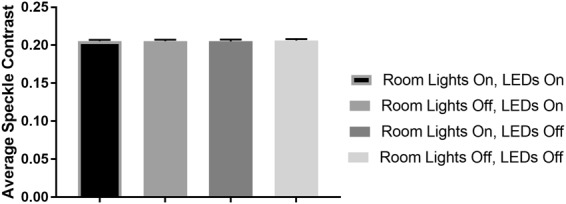
Average speckle contrast of LSCI images acquired using the LSCI-dermascope from a tissue phantom with room lights and tissue illumination LEDs on/off. Neither light source affected measurements collected with the LSCI-dermascope.

To determine whether room light and the white LED lights used for visual inspection of the skin would interfere with laser speckle contrast imaging (LSCI) measurements during LSCI-dermascope use, the average speckle contrast in a static tissue phantom was measured with and without these light sources on. The results can be seen in Fig. 1. The average speckle contrast did not vary significantly between any of the measurements (p > 0.05), demonstrating that neither room lights nor illumination LEDs significantly affected LSCI measurements.

### Quantification of measurement error due to user motion

**Figure 2:**
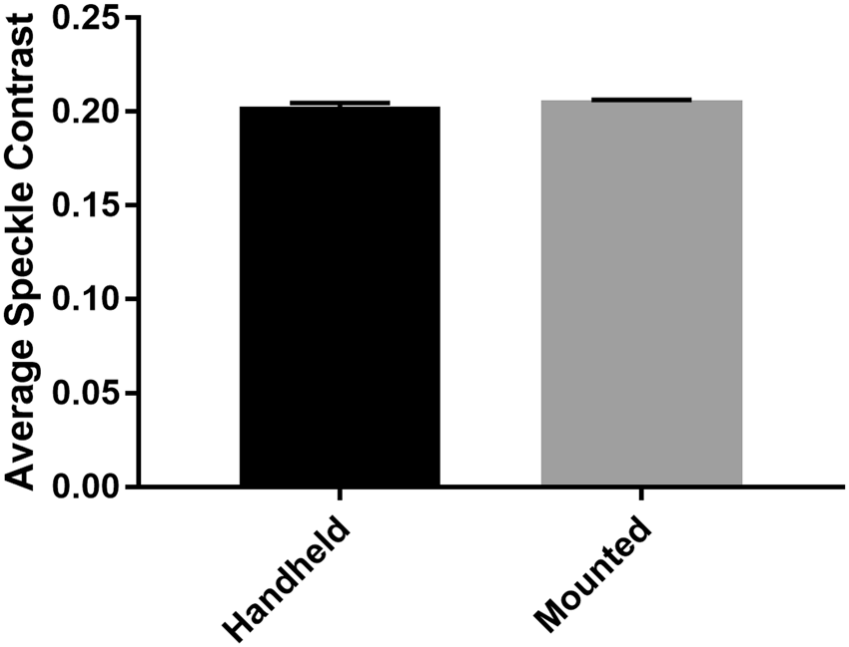
Average speckle contrast from a tissue phantom acquired from the LSCI-dermascope in a handheld and mounted configuration. These data suggest that handheld operation of the LSCI-dermascope did not affect measurement accuracy.

LSCI measurements are prone to artifact due to user movement. To quantify whether user movement significantly altered LSCI measurements performed using the LSCI-dermascope, average speckle contrast was measured from a static tissue phantom with the LSCI-dermascope either mounted or used handheld. The results can be seen in Fig. 2. The average speckle contrast did not vary significantly between mounted versus handheld measurements (p > 0.05), demonstrating that user movement during handheld use of the LSCI-dermascope did not significantly affect measurements relative to mounted use. Error bars represent standard deviation between measurements.

### Characterization of LSCI-dermascope measurements in a blood flow phantom

**Figure 3:**
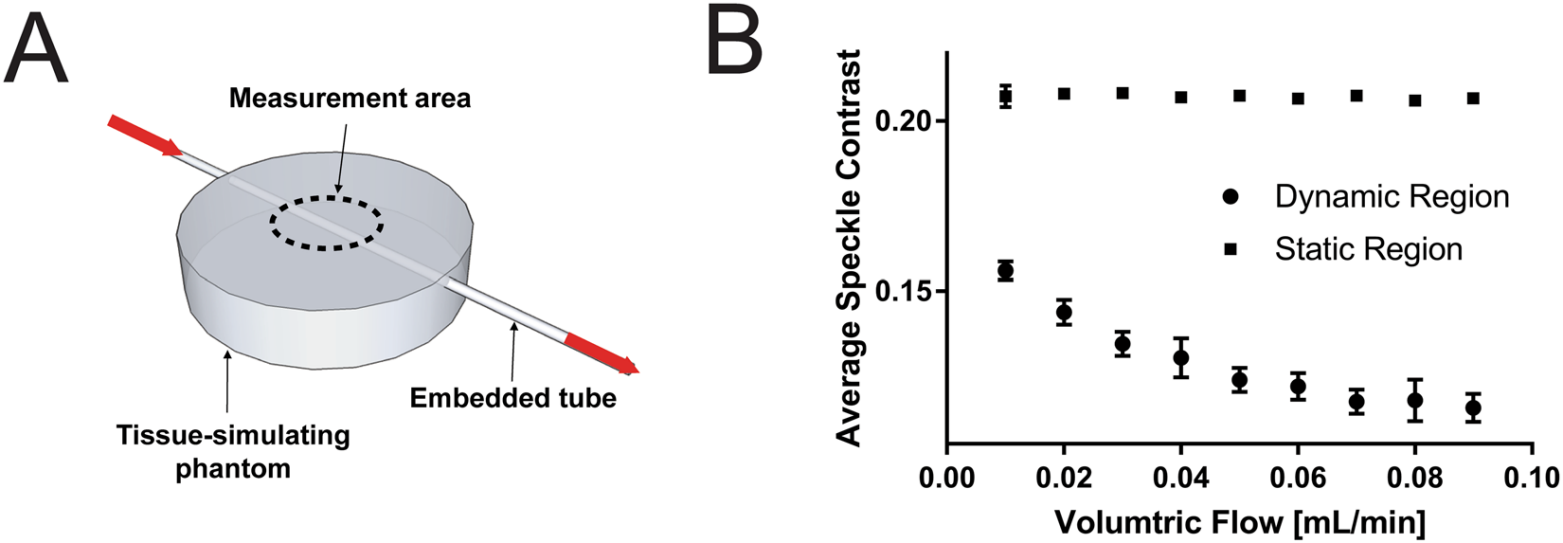
(**A**) Experimental setup used to characterize the response of LSCI measurements acquired with the LSCI-dermascope to varying flow rates within a simulated blood vessel embedded in a tissue phantom. The dotted line shows the approximate measurement area and location of the LSCI-dermascope cone. (**B**) Average speckle contrast within the area shown in (**A**) from a region above the tube (dynamic region) and a region not above the tube (static region) for known volumetric flows of Intralipid. Error bars represent standard deviation between measurements.

To characterize the response of LSCI-dermascope to varying degrees of simulated blood flow, LSCI measurements were acquired from a tissue phantom with an embedded tube through which Intralipid was flowed at known rates. A diagram of the measurement setup and plot of the average speckle contrast in a dynamic region above the tube and static region away from the tube for each volumetric flow are shown in Fig. 3. The decrease in speckle contrast relative to increasing volumetric flow in the dynamic region follows the expected exponential trend. The fact that the speckle contrast did not approach 0 at higher flow rates is likely due to the signal contribution from static scatterers around the embedded tube whose speckle contrast is not reduced by increasing flow rates within the tube. The speckle contrast in the static region did not significantly change as the volumetric flow rate was adjusted (p < 0.05). Error bars represent standard deviation between measurements.

### ***In vivo*** validation of LSCI-dermascope

**Figure 4:**
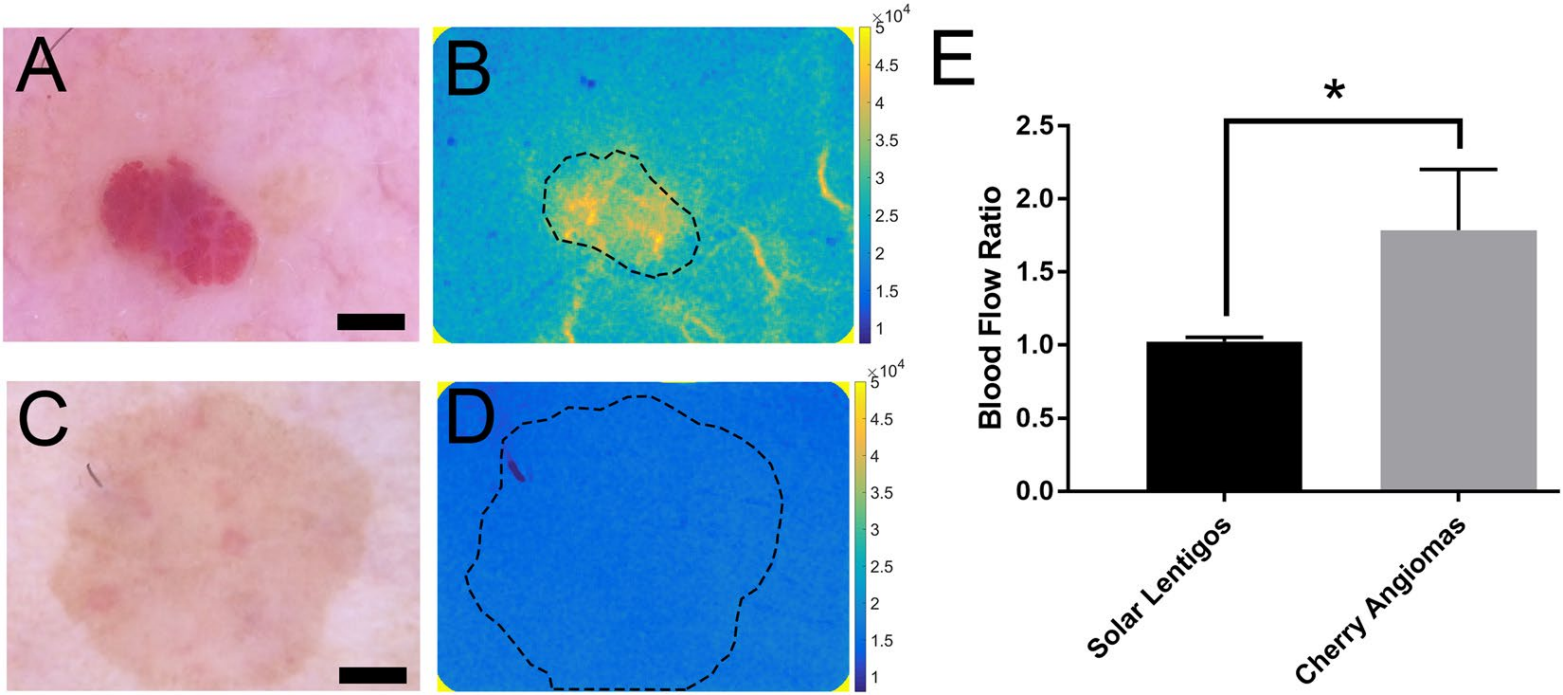
(**A**) Representative dermascope image of cherry angioma with corresponding blood flow map (**B**). (**C**) Representative dermascope image of solar lentigo with corresponding blood flow map (**D**). Scale bars in (**A**) and (**C**) are 1 mm. Dashed lines in (**B**) and (**D**) show region of interest selected for data averaging. Colorbars in (**B**) and (**D**) are in units of speckle flow index. (**E**) Average blood flow relative to surrounding normal skin for all LSCI-dermascope measurements collected from solar lentigos and cherry angiomas. Error bars represent standard deviation between average measurements.

The ability of the LSCI-dermascope to quantify differences in blood flow between different skin lesions was validated by acquiring LSCI measurements from lesions expected to have increased blood flow relative to the surrounding tissue (cherry angiomas, n = 13), and from skin lesions not expected to have increased blood flow relative to the surrounding tissue (solar lentigos, n = 10). Representative color images of each lesion type along with a corresponding blood flow map are shown in Fig. 4. Average blood flow within each lesion type relative to the surrounding skin is also shown in Fig. 4. The ratio of blood flow within cherry angiomas relative to surrounding skin was significantly higher than that of solar lentigos (p < 0.05). LSCI-dermascope measurements took approximately 10–15 seconds per lesion.

## Discussion

Dermascopes are one of the most frequently used tools by dermatologist for the inspection of suspicious skin lesions. However, while improvements to dermascopes over the past several decades have offered additional structural information, little to no functional information can be measured. Since abnormalities in functional tissue properties such as blood flow often accompany skin pathologies, the inability to measure such properties represents a potentially significant gap in diagnostic or prognostic capability. LSCI has been used extensively to measure tissue blood flow; however, its practical use in the dermatology clinic is limited by the fact that most measurement systems are relatively large and bulky, do not provide simple co-registration of inspected lesions with collected blood flow, and suffer from measurement artifact due to movement. We have described a LSCI-dermascope which addresses each of these limitations.

To address the size limitation of conventional LSCI systems, we utilized compact laser illumination and imaging hardware to create a device that is comparably sized to a conventional dermascope and equally simple to use. To address the difficulty in registering LSCI flow maps with visually inspected lesions, the LSCI-dermascope spectrally separates laser illumination from white-light illumination. We demonstrated that dermoscopy, even with the room lights on, could be performed simultaneously with LSCI measurements without significant effects on the collected data. To address the potential for measurement errors due to motion, we have developed a probe which uses minimal but direct contact with the subject to help stabilize the device. This resulted in LSCI measurements that could be acquired by hand without significant motion artifact.

Beyond the limitations above, we additionally addressed the relative nature of LSCI measurements by presenting blood flow information from measured lesions relative to that in the surrounding tissue. Acquiring LSCI data from both regions is relatively simple, because simultaneous imaging with white light streamlines identification of lesions and their borders. By utilizing the surrounding tissue of an inspected lesion as an internal control, factors that can affect LSCI measurements but unrelated solely to lesion blood flow can be minimized or eliminated. These include changes in laser coherence, imaging angle, tissue curvature, room temperature, subject position, and others^25–29^. As such, utilizing an internal control for measurements is potentially useful for collecting data that can be compared from subject to subject.

Finally, in a small pilot clinical study, we demonstrated the ability of the LSCI-dermascope to measure significant blood flow differences between two distinct skin lesion types: cherry angiomas and solar lentigos. This result is consistent with expected results. Cherry angiomas are papules with an abnormally high density of superficial capillaries that are expected to exhibit increased perfusion relative to normal skin. Alternatively, solar lentigos are macules of the epidermis characterized by increased basal layer pigmentation without alterations in the skin microvasculature and therefore are not expected to exhibit altered perfusion relative to the surrounding skin^30–34^.

In summary, we have presented a novel dermascope that is capable of providing functional tissue information in combination with standard structural information. By addressing many of the shortcomings associated with typical LSCI measurements, the LSCI-dermascope enables relatively quick and simple measurements perfusion measurements from lesions. Very little additional time is required for LSCI-dermascope measurements compared to conventional dermoscopy, facilitating practical use in the clinic. Future study will entail determining whether lesion blood flow information provided by the LSCI-dermascope can be used adjunctively with standard lesion diagnostic assessment to improve diagnostic accuracy without necessitating tissue biopsy.

## Methods

### Construction of LSCI-dermascope

**Figure 5:**
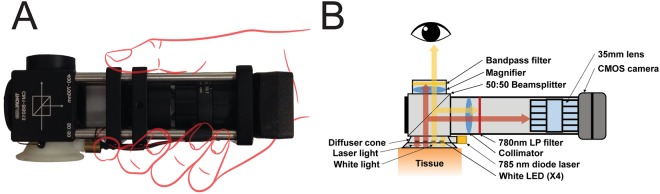
(**A**) Photograph of the LSCI-dermascope. (**B**) Cutaway drawing of the LSCI-dermascope illustrating the internal components that enable simultaneous LSCI measurements and conventional dermoscopy.

A photograph of the LSCI-dermascope and a diagram illustrating the internal components is shown in Fig. 5.

Conventional dermascope functionality was performed by illuminating the tissue under inspection with four white LEDs (SMLP12WBC7W1, Rohm Semiconductor, Kyoto, Japan) wired in series with a 20 mA current limiter (NSI45020, ON Semiconductor, Phoenix, Arizona) and mounted inside of a light diffusing cone that was placed in direct contact with the skin during imaging. LED light remitted from the tissue entered a 50:50 beam splitter (CM1-BS013, Thorlabs Inc., Newton, New Jersey) that directed approximately half of the light into the handle of the LSCI-dermascope and approximately half of the light toward the eye of the user. LED light directed into the handle was collimated using a 35 mm focal length lens (59–872, Edmund Optics Inc., Barrington, New Jersey) and then attenuated using a 780 nm longpass dielectric filter (ET780lp, Chroma Inc., Bellows Falls, Vermont). LED light directed toward the eye of the user was magnified using a 60 mm focal length lens (LB1596-A, Thorlabs Inc., Newton, New Jersey) and then passed through a 700 nm shortpass filter (FESH0700, Thorlabs Inc., Newton, New Jersey).

To perform LSCI simultaneously with conventional dermascope imaging, a 50 mW 785 nm laser diode (785MD-3-0610, LaserLands, Spalstraat, Netherlands) was affixed to the side of the diffusing cone. The diffusing cone was machined from an acetal polymer whose optically scattering properties created a more homogenous illumination field for LSCI measurements than would be achieved by illuminating the tissue directly by the laser diode^35^. Laser light remitted from the same region of tissue being imaged during conventional dermascope measurements was also separated using the 50:50 beam splitter. Laser light directed toward the eye of the operator was attenuated via the shortpass filter. Laser light directed toward the handle of the device was collimated using a 35 mm focal lens and then directed through a 780 nm longpass filter and into a 35 mm camera lens mounted to a CMOS camera (FireFly MV, 6.0 µm pixel size, FLIR Integrated Imaging Solutions, Inc., BC, Canada). The 3.3 V power supply on the CMOS camera was used to power both the LEDs and laser diode during imaging. The camera lens was set to an f/# of 8, as this aperture enabled sampling of the speckle pattern which satisfied the Nyquist criterion^36^. The resulting dual-mode imaging system enabled acquisition of co-registered visual and camera-based (LSCI) images.

### Flow measurements using the LSCI-dermascope

Quantitative blood flow maps were acquired using LSCI, which is an optical technique that uses a coherent light source and a CMOS/CCD camera to create wide-field quantitative maps of tissue blood flow^37–41^. The technique is based on the principle of quantifying the spatiotemporal changes in the speckle pattern that result from the constructive and destructive interference of the laser light used to illuminate the tissue. Movement of blood within the tissue causes changes in the speckle pattern and blurring in collected images, which can be quantified using a metric called speckle contrast to create full-field maps of tissue blood flow. Speckle contrast, *K*, is conventionally computed as:

1 $$K=\frac{\sigma }{ < I > }$$

where *σ* and <*I>* represent the standard deviation and average number of the pixel counts in a local neighborhood of pixels. LSCI images acquired with the LSCI-dermascope were acquired at a rate of 30 frames per second and at a pixel resolution of 640 × 480. Speckle contrast maps were computed from raw images using a 7 × 7 spatial sliding window algorithm that implemented Equation (1). In some instances, speckle contrast maps were converted to speckle flow index (SFI) maps because SFI has been shown to correlate linearly with blood flow and therefore offers a more intuitive representation of flow within an object^42,43^. SFI was computed as:

2 $$SFI=\frac{1}{2T{K}^{2}}$$

where *T* represents the exposure time of the camera using during image acquisition. In all experiments, an exposure time of 5 ms was used^44^. Image acquisition and processing was performed using custom software written in MATLAB.

### Quantification of LED/room light interference on LSCI measurements

The presence of LED or room light within images collected for LSCI measurements can potentially introduce measurement error. Room light which travels through the lens of the LSCI-dermascope and white LED light projected directly onto the region of interest are relatively broadband sources incoherent light, which if not adequately filtered from the signal detected by the camera system, will result in an artificial decrease in speckle contrast. To quantify this effect on the LSCI-dermascope, LSCI measurements were acquired from a tissue-simulating phantom with and without the LEDs and fluorescent room lights turned on. The phantom was fabricated from polydimethylsiloxane with titanium dioxide added to achieve a reduced scattering coefficient of 1 mm^−1^ at 785 nm^45,46^. For each measurement condition (LEDs and room lights on/off), 10 measurements were acquired and averaged, each of which was derived from an average of 30 speckle contrast maps.

### Quantification of measurement error due to user motion

Movement during LSCI can cause significant artifacts and measurement error. To quantify the effect of motion artifact imparted from using the LSCI-dermascope in a handheld fashion as opposed to mounted, LSCI measurements were acquired under these conditions from the tissue phantom used above. During handheld measurements, the LSCI-dermascope was held in direct contact with the tissue phantom for five seconds, during which 30 images were collected. Between measurements, the LSCI-dermascope was removed from the tissue phantom and then replaced. The same protocol was followed for mounted measurements but with the LSCI-dermascope rigidly mounted during image acquisition. For each condition, 10 measurements were acquired and averaged, each of which was derived from an average of 30 speckle contrast maps.

### Characterization of LSCI-dermascope measurements in a blood flow phantom

The response of the LSCI-dermascope to known fluid flow rates was characterized by acquiring data from a tissue phantom with an embedded tube to simulate a subsurface blood vessel which was located 0.5 mm below the surface. The phantom was fabricated from polydimethylsiloxane with titanium dioxide added to achieve a scattering coefficient of 1 mm^−1^ at 785 nm^45,46^. The embedded tube was made of Tygon and had an inner diameter of 0.79 mm. A syringe pump was used to flow lipid emulsion (Intralipid 20%, Fresenius Kabi, Toronto, Canada) at volumetric flow rates ranging from 0.01 to 0.09 mL/min. This range was chosen to simulate the approximate speed of blood flow in skin capillaries^47^. Intralipid was diluted to a concentration of 0.54% to result in a reduced scattering coefficient of 0.6 mm^−1^, which is comparable to that of human blood at 785 nm^48–51^. For each volumetric flow rate, the LSCI-dermascope was placed at the location shown in Fig. 3A and 30 images acquired. The average speckle contrast within 50 × 200 pixel region of each image corresponding to the area above the embedded tube (the dynamic region) and a 50 × 200 pixel region not above the embedded tube (the static region) was computed and averaged between all 30 frames.

### ***In vivo*** validation of LSCI-dermascope

To assess the LSCI-dermascope’s ability to differentiate quantitatively between skin lesions with suspected blood flow differences, LSCI data were acquired from 13 cherry angiomas and 10 solar lentigos. Cherry angiomas were expected to exhibit increased blood flow relative to the surrounding skin, whereas solar lentigos were not. Diagnosis of each lesion type was performed by a trained dermatologist (KMK) at the University of California, Irvine Health Gottschalk Medical Center. During LSCI measurements, the dermascope was placed over the lesion of interest and proper location confirmed visually using the magnified white-light image. Fifty images were acquired from each lesion. During processing, each image was converted to a SFI map and then averaged together. The median SFI was computed within a manually-selected region of interest corresponding to the lesion and the surrounding normal skin. A blood flow ratio was then computed for each lesion by dividing the median SFI in the lesion by the median SFI within the surrounding skin. The average of these ratios was computed for all cherry angiomas and for all solar lentigos. All human data collection was collected in accordance with methods approved by the University of California Institutional Review Board (HS#2008-6307). Informed consent was obtained from all subjects.

### Statistical Analysis

Statistical differences among populations were analyzed using either ANOVA or the Mann-Whitney test. p-values less than 0.05 were considered significant.

## Data Availability

The datasets generated during and/or analyzed during the current study are available from the corresponding author on reasonable request.
